# Functions and Clinical Applications of Extracellular Vesicles in T_H_2 Cell-Mediated Airway Inflammatory Diseases: A Review

**DOI:** 10.3390/ijms25179455

**Published:** 2024-08-30

**Authors:** Jaehwan Cheon, Byoungjae Kim, Juhyun Lee, Jaemin Shin, Tae Hoon Kim

**Affiliations:** 1 Department of Biomedical Science, Korea University College of Medicine, Seoul 02841, Republic of Korea; 2 Department of Otorhinolaryngology-Head & Neck Surgery, Korea University College of Medicine, Seoul 02841, Republic of Korea; 3 Neuroscience Research Institute, Korea University College of Medicine, Seoul 02841, Republic of Korea; 4 Mucosal Immunology Institute, Korea University College of Medicine, Seoul 02841, Republic of Korea

**Keywords:** type 2 inflammation, extracellular vesicles, chronic rhinosinusitis with nasal polyp, allergic rhinitis, asthma

## Abstract

Type 2 airway inflammation (T2AI), driven by type 2 innate lymphoid and CD4^+^ T helper 2 cells, leads to various diseases and conditions, such as chronic rhinosinusitis with nasal polyps, allergic rhinitis, and asthma. Emerging evidence suggests the involvement of extracellular vesicles (EVs) in these diseases. In this review, we describe the immunological T2AI pathogenic mechanisms, outline EV characteristics, and highlight their applications in the diagnosis and treatment of T2AI. An extensive literature search was conducted using appropriate strategies to identify relevant articles from various online databases. EVs in various biological samples showed disease-specific characteristics for chronic rhinosinusitis with nasal polyps, allergic rhinitis, and asthma, with some demonstrating therapeutic effects against these conditions. However, most studies have been limited to in vitro and animal models, highlighting the need for further clinical research on the diagnostic and therapeutic applications of EVs.

## 1. Introduction

Type 2 airway inflammation (T2AI) is characterized by an immune response mediated by type 2 innate lymphoid cells (ILC2s) and a subset of CD4^+^ T helper 2 (T_H_2) cells in the respiratory system. The “one-airway-one-disease” hypothesis postulates that the upper and lower airways share a similar pathophysiology [1]. Therefore, excessive type 2 inflammation (T2I) is able to lead to chronic rhinosinusitis (CRS), allergic rhinitis (AR), and asthma depending on its location in the airway tract. Over the past 5 years, the global prevalence of type 2 airway diseases has steadily increased, with CRS, AR, and asthma affecting approximately 5–12% [2], 10–30% [3], and 3–10% [4] of the global population, respectively. These diseases exhibit correlated characteristics under airway inflammatory conditions [5]. For example, individuals with asthma often exhibit AR and CRS symptoms [6], whereas those with CRS commonly exhibit asthma as a comorbidity [7]. Despite advancements in diagnosis and medication, treatment remains challenging, attributable to the complexity of these comorbid conditions, which share a similar pathogenesis in the upper and lower airway tracts [1,2,8,9]. The overlapping symptoms and inflammatory pathways in CRS, AR, and asthma further complicate their diagnosis and treatment, necessitating additional research and improved clinical strategies for managing these conditions.

Recent studies have increasingly highlighted the diagnostic and therapeutic applications of extracellular vesicles (EVs) for various diseases [10,11,12]. EVs, vesicles derived from cellular membranes, transport biomacromolecules to recipient cells. As EVs act as carriers of cargo molecules, they are considered important mediators of cellular communication [13]. Owing to their heterogeneity, which is influenced by the conditions of the secreting cells and their role in delivering cargo molecules to specific targets, EVs are considered promising agents for the diagnosis and therapy of various diseases [14].

In this review, we discuss the intricate pathogenesis of T2AI and outline the characteristics of EVs. Furthermore, we highlight the current potential applications of EVs for the diagnosis and treatment of T2AI-related diseases. We summarize recent research findings that demonstrate the potential of EVs as biomarkers for early disease detection and as therapeutic agents capable of modulating immune responses and inhibiting inflammation. We provide a comprehensive overview of the functions and clinical applications of EVs in T2AI diseases and outline future research directions to facilitate and advance effective diagnostic and therapeutic strategies.

## 2. The Pathogenesis of T2AI Diseases

The airway is anatomically divided into the upper respiratory tract, including the nasal cavity, pharynx, and larynx, and the lower respiratory tract, including the trachea, bronchi, and bronchioles [15]. Excessive T2I predominantly causes CRS and AR in the paranasal sinuses and turbinates, whereas asthma primarily affects the lower respiratory tract. The airway is a tract largely composed of mucosa, a tissue comprising various epithelial cells that primarily regulate immune responses by serving as physical barriers [16]. Various genetic and environmental factors can stimulate this epithelial layer, leading to barrier disruption and induction of T2I [17,18].

### 2.1. T2I Occurs via Interactions among the Epithelial Cells, Dendritic Cells, and T-Cells

Disrupted epithelial cells release chemokines, including C-C motif chemokine ligands (CCLs), and alarmin cytokines, such as the thymic stromal lymphopoietin (TSLP), interleukin (IL)-25, and IL-33 [18,19]. These chemokines and cytokines not only activate ILC2s but also attract dendritic cells (DCs) expressing C-C motif chemokine receptors to the site of disruption. At this location, immature DCs (imDCs) are converted into mature DCs (mDCs) via stimulation by alarmin cytokines and the binding of antigens and allergens to pattern recognition receptors on the surface membrane of DCs [18]. This maturation process is crucial for the initiation and propagation of immune responses, since mDCs function as key antigen-presenting cells [20]. They express major histocompatibility complex class II (MHC II) molecules, and the processed antigens or allergen peptide fragments are presented on these molecules. This presentation is essential for T-cell activation and adaptive immune responses [21]. Activated mDCs migrate to the local lymph nodes, where they polarize naïve T cells into T_H_2 cells by forming an immune synapse with T-cells. The binding of antigen or allergen peptide fragment-loaded MHC II to the T-cell receptor (TCR) provides the primary signal necessary for T-cell polarization [22]. This interaction leads to the differentiation and expression of antigen- or allergen-specific T_H_2 cells, which are essential for adaptive immune responses [23].

### 2.2. T_H_2 Cells Trigger Adaptive Immune Responses by Interacting with B-Cells

Polarized T_H_2 cells in the lymph nodes migrate to the mucosa, where they are activated by alarmin cytokines derived from epithelial cells and ILC2s. Once activated, both ILC2s and T_H_2 cells release the signature T_H_2 cytokines, IL-4, IL-5, and IL-13 [24]. These cytokines play critical roles in immune responses by inducing B-cells to undergo immunoglobulin E (IgE) class switching during their interactions with T_H_2 cells [25]. Activated B-cells differentiate into plasma cells, which secrete IgE and memory B-cells, facilitating a rapid adaptive immune response to specific antigens or allergens [26].

### 2.3. T2I Is Initiated by Innate Immunity Activation by T_H_2 and B-Cells

T_H_2 cytokines secreted by ILC2s and T_H_2 cells affect epithelial cells, granulocytes, and B-cells. IL-4 induces the transformation of epithelial cells into goblet cells, which primarily produce the mucus [27]. IL-5 activates granulocytes, particularly eosinophils, leading to their degranulation [28]. The stimulated eosinophils degranulate, causing mucus thickening and increasing epithelial damage [27,29].

**Figure 1:**
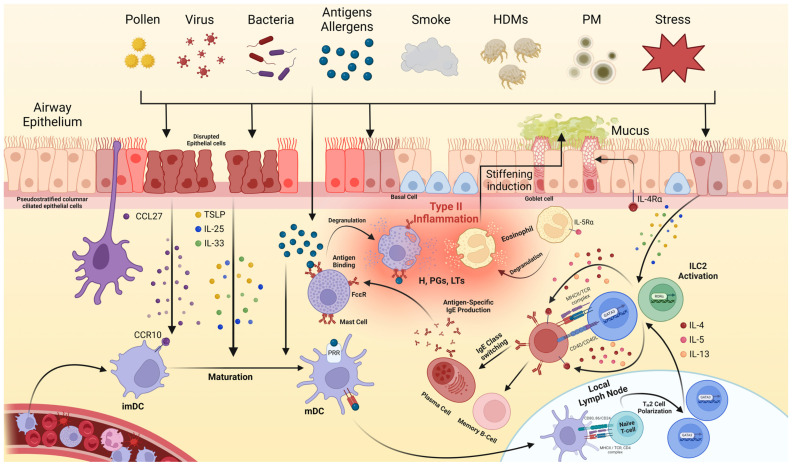
The pathogenesis of type 2 airway inflammation (T2AI). Various triggers, including pollens, viruses, bacteria, antigens/allergens, smoke, house dust mites (HDMs), particulate matter (PM), and stress, disrupt the airway epithelium, leading to the release of chemokines, such as the C-C motif chemokine ligand (CCL)-27, and alarmin cytokines, such as thymic stromal lymphopoietin (TSLP), interleukin (IL)-25, and IL-33. These signals activate type 2 innate lymphoid cells (ILC2s) and attract dendritic cells (DCs) expressing chemokine receptors, such as the C-C motif chemokine receptor (CCR)-10, to the site of disruption. Immature DCs (imDCs) migrate to the disrupted epithelium and are converted to mature DCs (mDCs) via stimulation by alarmin cytokines and interactions with the infected antigens/allergens. These mDCs present the antigens to naïve T-cells in local lymph nodes, promoting their polarization into T_H_2 cells. These T_H_2 cells, along with ILC2s, secrete cytokines (IL-4, IL-5, and IL-13), which influence various cell types, such as epithelial cells and eosinophils. Additionally, IL-4 and IL-13 facilitate B-cell class switching to IgE. Plasma cells secrete IgE, which binds to the IgE receptors (FcεR) on mast cell membranes via a process known as sensitization. Upon re-exposure to the antigen or allergen, the cross-linking of IgE on mast cells induces their degranulation, releasing inflammatory mediators, such as histamines (Hs), prostaglandins (PGs), and leukotrienes (LTs). Additionally, IL-5 activates and degranulates eosinophils. This integrative degranulation of mast cells and eosinophils ultimately leads to inflammatory symptoms in the airway. HDM, house dust mite; PM, particulate matter; CCL, C-C motif chemokine ligand; TSLP, thymic stromal lymphopoietin; IL, interleukin; ILC2, type 2 innate lymphoid cell; DC, dendritic cell; CCR, C-C motif chemokine receptor; imDC, immature DC; mDC, mature DC; Ig, immunoglobulin; FcεR, IgE receptor; H, histamine; PG, prostaglandin; LT, leukotriene.

IgE released from plasma cells binds to the IgE receptor (FcεR) on mast cell membranes through a process known as sensitization in allergic responses. As IgE bound to mast cells is specific to certain antigens or allergens, when these antigens or allergens re-enter the disrupted epithelial cells, they directly bind to the IgE on mast cells. This cross-linking triggers mast cell degranulation, releasing inflammatory molecules, such as histamines, prostaglandins, and leukotrienes [30]. These factors ultimately induce inflammatory symptoms, such as coughing, sneezing, and airway congestion [17]. Figure 1 illustrates the overall immunological pathogenic mechanism of T2AI.

## 3. EVs

### 3.1. The Biogenesis of EVs

EVs are secreted by donor cells through various biological processes and they are primarily classified into apoptotic bodies (ApoBDs), micro-vesicles, and exosomes based on their cell of origin and vesicle size [31,32,33]. ApoBDs (diameter: 1–5 µm) are vesicles shed from apoptotic cells during programmed cell death. They contain proteins, lipids, and genetic materials, such as mRNAs, micro-RNAs (miRNAs), and other cellular organelles [31]. These vesicles are formed by a tightly regulated process, called apoptotic cell disassembly, which involves plasma membrane blebbing, formation of apoptotic protrusions, and fragmentation into ApoBDs [34]. ApoBDs are produced by dying cells, whereas micro-vesicles and exosomes are formed via the budding or exocytosis of living cells.

Micro-vesicles are formed by the outward budding of the cellular membrane, leading to the detachment and formation of vesicles larger than 200 nm in diameter. Membrane budding occurs at specific sites on the plasma membrane and is influenced by phospholipid redistribution. This mechanical redistribution, along with the phosphorylation of ρ-kinase-mediated myosin light chain and contractile machinery, facilitates the pinching and detachment of vesicles [35,36]. Unlike ApoBDs, micro-vesicles do not contain cellular organelles but do include proteins, lipids, and genetic materials. The cytosolic biomolecules are incorporated randomly, whereas membrane proteins and receptors are specifically directed to the plasma membrane before micro-vesicle budding. Micro-vesicles might be identified by their lipid composition, plasma membrane receptors, and biomolecular content, which reflect their cellular origin [32]. 

Similar to micro-vesicles, exosomes are vesicles that contain proteins, lipids, and genetic materials; however, they have a more complex and heterogeneous formation mechanism than micro-vesicles and are typically less than 150 nm in diameter. They are mainly classified into classical and non-classical exosomes (exosome-like vesicles). Classical exosomes are derived from the endosomal sorting complex required for the transport (ESCRT)-dependent pathway. This pathway involves the inward invagination of the endosomal membrane facilitated by ESCRT proteins, such as ESCRT-0 (hepatocyte receptor tyrosine kinase substrate [HRS], also known as the vacuolar protein sorting-associated protein [VPS]-27), ESCRT-I (tumor susceptibility gene 101 [TSG101]), ESCRT-II (VPS25), and ESCRT-III (charged multivesicular body protein [CHMP]-4A, CHMP4B, and CHMP4C) [37]. These proteins recruit accessory proteins, such as the ALG-2-interacting protein X, leading to the formation of multivesicular bodies (MVBs) that contain several intraluminal vesicles (ILVs) [38]. MVBs are capable of being formed via an ESCRT-independent pathway involving sphingomyelinase hydrolysis and ceramide formation [39]. During MVB formation, ILVs package endosomal or cytosolic proteins and genetic materials that are reflective of their parental cells. Subsequently, tetraspanins proteins, such as CD9, CD63, and CD81, are incorporated into MVBs, as they are associated with recycling routes between the plasma membrane and various cellular organelles [40]. MVBs are produced via either ESCRT-dependent or-independent mechanisms and subsequently fuse with the cellular membrane to release ILVs, which become exosomes or exosome-like vesicles [41].

Synthesized and released from donor-cells, EVs are capable of inducing various intra-cellular alterations of recipient cells. For example, When RNA contained within EVs is delivered to target cells, it contributes to gene expression, leading to changes in protein synthesis within the recipient cells [42,43]. Also, EVs carrying miRNAs are able to suppress the expression of their target genes within recipient cells [44].

### 3.2. The Classification of EVs

**Figure 2:**
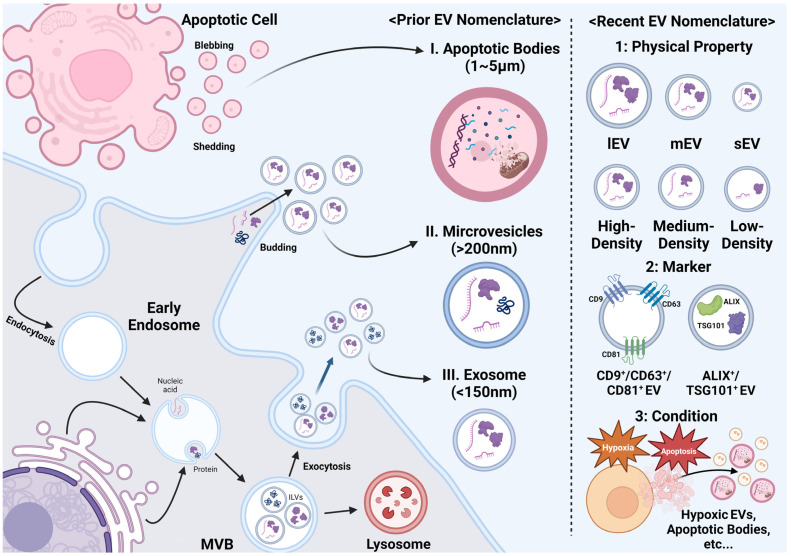
The biogenesis and Classification of extracellular vesicles (EVs). EVs are classified into apoptotic bodies (ApoBDs), micro-vesicles, and exosomes based on their origin and size. ApoBDs are primarily formed via the shedding and blebbing of apoptotic cells, whereas micro-vesicles are produced via cellular budding. Exosomes are typically formed and released through the endosomal sorting complex required for the transport (ESCRT)-dependent pathway, with markers, such as tetraspanins, the tumor susceptibility gene 101 (TSG101), and the ALG-2-interacting protein X (ALIX), playing key roles in their identification. Recently, EV classification has become more nuanced, taking into account their size, density, biochemical composition, and condition of parent cells. EV, extracellular vesicle; ApoBD; apoptotic body; ESCRT, endosomal sorting complex required for transport; TSG, tumor susceptibility gene; ALIX, ALG-2-interacting protein X; lEV, large EV; mEV, medium EV; sEV, small EV.

EVs are primarily classified into ApoBDs, micro-vesicles, and exosomes. Accurately categorizing EVs is challenging because of their potential to carry markers common to multiple biogenic pathways prior to secretion. The International Society for Extracellular Vesicles has provided comprehensive guidelines on characterizing EVs and assessing their purity [45]. The Minimal Information for Studies of EVs 2018 recommends the use of recent EV nomenclature to categorize EVs based on various criteria, including physical properties, specific markers, and the conditions or cells of origin. This approach aims to standardize EV research and improve the reproducibility and accuracy of findings in the field. Figure 2 illustrates the overall process of the generation and classification of EVs.

### 3.3. The Detection and Analysis of EVs

Recently, several techniques for detecting and analyzing EVs from biological samples have been developed, including ultracentrifugation, size-exclusion chromatography, nanoparticle tracking analysis (NTA), flow cytometry, and Western blotting (WB). Since each separation method has its own unique advantages and limitations, it is crucial to understand their specific characteristics and select the most suitable technique for the sample at hand to ensure highly reproducible results.

## 4. The Applications of EVs for Various T2AI Diseases

Among the various EVs subtypes, exosomes or small EVs (sEVs) are the most commonly utilized and applied in various diseases [46]. Similar to exosomes, sEVs measure between 30 and 150 nm in diameter and constitute the most prevalent subset of EVs in biological fluids [47]. In the case of T2AI diseases, the majority of research has also focused on the use of exosomes or sEVs.

### 4.1. The Application of EVs for CRS

CRS is a complex inflammatory disease affecting the mucosa of the nasal cavity and paranasal sinuses in the upper airway tract. Common symptoms of CRS include facial pain or pressure, nasal discharge, congestion, and a reduced sense of smell (hyposmia) or a complete loss of smell (anosmia). It significantly impacts quality of life and poses a substantial economic burden on affected patients. Traditionally, CRS is categorized based on the presence/absence of nasal polyps as CRSsNPs (without nasal polyps) and CRSwNPs (with nasal polyps) [2,48]. Patients with CRSwNP are more likely to experience concurrent lower airway inflammation, such as asthma, than those with CRSsNP. Compared with those with CRSsNP, patients with CRSwNP typically exhibit more severe symptoms, require more frequent surgeries, and have higher recurrence rates after surgical intervention. These factors are crucial considerations for their effective clinical treatment [49]. CRSwNP arises from heterogeneous conditions and is primarily classified into type 2 and non-type 2 endotypes. Type 2 CRSwNP, common in Western countries, is mediated by T2I, whereas non-type 2, including types 1 and 17, is mediated by T_H_1 and T_H_17 cells and their signature cytokines, interferon (IFN)-γ and IL-17, respectively [50]. Recently, CRSwNP has been further classified into eosinophilic CRSwNP (eCRSwNP) and non-eCRSwNP based on the histological quantification of eosinophil numbers [51]. ECRSwNP is strongly associated with T2I and worse clinical prognosis than non-eCRSwNP [52,53]. Consequently, many studies have focused on CRSwNP mediated by T2I or eCRSwNP to improve patient treatment outcomes.

Recently, numerous studies have focused on detecting altered biomacromolecules in patients with CRSwNP compared to biomolecules in the normal groups using EVs derived from various biological samples. In the mucus, expression of cystatin (CST)-2, also known as CST-SA, is significantly higher in the nasal mucus-derived EVs (NMDEs) of patients with CRSwNP than in those of patients with CRSsNP and controls. Moreover, CST-2 expression is closely related to tissue eosinophilia, asthma, and allergies [54]. This finding is supported by previous studies indicating that CSTs, which are endogenous protease inhibitors, play crucial roles in maintaining the epithelial barrier and modulating immune responses. CSTs also contribute to the recurrence of eosinophilic CRS by interacting with various components, including T_H_2 cytokines and fibroblasts [55,56]. Mueller et al. reported that the expression of specific serpin family members, including SerpinB2, SerpinE1, SerpinF2, and SerpinG1, are up-regulated in both the matched tissues (CRSwNP: middle turbinate polypoid mucosa; control: inferior turbinate) and NMDEs of patients with CRSwNP compared with their expression in the controls [57]. These serpins inhibit the fibrinolysis pathway by inactivating the tissue plasminogen activator, urokinase plasminogen activator, and plasmin. Therefore, increased expression of the serpin family in CRSwNP possibly contributes to polyp formation by maintaining fibrin deposition [57]. Moreover, pappalysin-A (PAPP-A) levels were significantly increased in both the matched tissues (CRSwNP: adjacent nasal polyp/control: middle turbinate) and NMDEs of patients with CRSwNP. PAPP-A cleaves its substrates, such as insulin-like growth factor (IGF) binding proteins (IGFBP)-4/5, which inactivate IGFs, thereby leading to the activation of IGFs. Therefore, up-regulation of the PAPP-A/IGFBP-4/5/IGF-1 axis in CRSwNP is possibly associated with enhanced epithelial proliferation and polyp growth [58].

Differential expression of specific targets in CRSwNP has been reported in blood samples. He et al. reported that patients with CRSwNP exhibit differentially expressed miRNAs (DEMs) in plasma, with increased levels of miR-79, miR-677, and miR-1037 and decreased levels of miR-4, miR-192, and miR-1022 compared to the controls [59]. miRNAs are non-coding RNAs, approximately 21–24 nucleotides long, that play post-transcriptional regulatory roles by binding to target mRNAs, leading to mRNA degradation or the inhibition of protein translation [60]. These DEMs in CRSwNP plasma are involved in various pathways, such as extracellular matrix–receptor interactions, calcium signaling, and the Hippo, Notch, ErbB, and cAMP signaling pathways [54]. Moreover, galectin 10 (GAL10) and eosinophil peroxidase (EPO) levels in both the tissues (CRSwNP: nasal polyp/CRSsNP: inferior turbinate) and serum EVs are higher in patients with CRSwNP than in those with CRSsNP [61]. Galectins are a family of 15 lectins implicated in various biological processes, including immune responses, inflammation, cell proliferation, motility, and programmed cell death [62]. EPO, a heme protein that is abundantly expressed in eosinophils, catalyzes the production of cytotoxic oxidants and contributes to the pathogeneses of cancer, asthma, and allergic inflammatory disorders [63]. Notably, GAL10 in the EVs released from eosinophils is possibly associated with the development of CRSwNP by promoting eosinophil extracellular trap cell death, which leads to sinus obstruction [61].

EVs of patients with CRSwNP are able to be detected and analyzed using biological fluids, such as mucus and blood samples, and primary cells. Zhou et al. demonstrated that the primary human nasal epithelial cells (hNECs) of patients with CRSwNP contain differentially expressed proteins (DEPs) in their derived exosomes. These patients exhibit increased levels of integrin-β5, endoplasmic reticulum membrane protein complex subunit 4, L1 cell adhesion molecule, and transaldolase 1 and decreased levels of torsin family 4 member A, small proline-rich protein 3, and solute carrier family 1 member 1 compared to the healthy controls. These DEPs are mainly involved in epithelial remodeling. Notably, exosomes derived from the epithelial cells of patients with CRSwNP significantly inhibit the proliferation rate of normal hNECs [64]. The inhibition of adjacent normal cells by DEPs in EVs derived from the hNECs of patients with CRSwNP may be due to the activation of the p53 signaling pathway, leading to the suppression of proliferation [64]. Table 1 provides a detailed overview of EVs investigated in recent CRSwNP studies.

### 4.2. The Application of EVs for AR

AR occurs via IgE-mediated responses to inhaled allergens, leading to mucosal inflammation driven by T2I in the upper airway. It is characterized by various symptoms, such as sneezing, nasal congestion, nasal itching, and rhinorrhea (nasal discharge). AR is a prevalent chronic condition, especially in high-income countries, where its prevalence can reach up to 50%, but it is relatively rare in low- and middle-income countries [65]. AR is a significant global health concern that contributes substantially to the overall burden of disease and disability worldwide. Moreover, AR is associated with decreased productivity at work and school, sleep disturbance, and reduced participation in outdoor activities among children [66,67].

**Table 1:** List of studies investigating extracellular vesicles (EVs) in chronic rhinosinusitis with nasal polyps (CRSwNP).

No.	EV Donor	Used EVs	EV Isolation Methods	EV Identification Methods	Subjects	Results/Effects	Ref.
1	Mucus	Exosomes/sEVs	Ultracentrifugation	Not available	Patients with CRSwNP vs. Control	CST-2 (CST-SA) ↑	[54]
2	Mucus	Exosomes/sEVs	Centrifugation (1500× *g* for 30 min, 12,000× *g* for 45 min) Ultracentrifugation (110,000× *g* for 2 h) Filtration with 0.22-µm filter Centrifugation (110,000× *g* for 70 min)	Not available	Patients with CRSwNP vs. Control	SerpinB2, SerpinE1, SerpinF2, SerpinG1 ↑	[57]
3	Mucus	Exosomes/sEVs	Centrifugation (1500× *g* for 30 min, 12,000× *g* for 45 min) Ultracentrifugation (110,000× *g* for 2 h) Filtration with 0.22-µm filter Centrifugation (110,000× *g* for 70 min)	Not available	Patients with CRSwNP vs. Control	PAPP-A ↑	[58]
4	Plasma	Exosomes/sEVs	Centrifugation (5000× *g* for 20 min) Filtration with 0.45-µm filter Size exclusion column	NTA (100 nm) WB (CD9, CD63, TSG101, ALIX) TEM (40–160 nm)	Patients with CRSwNP vs. Control	miR-677, miR-1037, miR-79 ↑ miR-192, miR-1022, miR-4 ↓	[59]
5	Serum	Exosomes/sEVs	PS affinity method	TEM (CD9) NTA (<200 nm)	Patients with CRSwNP vs. CRSsNP	GAL10, EPO ↑	[61]
6	Epithelial cell	Exosomes/sEVs	Centrifugation (1000× *g* for 10 min, 16,500× *g* for 30 min) Ultracentrifugation (100,000× *g* for 2 h)	NTA TEM WB (CD9, TSG101)	Patients with CRSwNP vs. Control EV treatment after vs. before into normal hNEC	ITGB5, EMC4, L1CAM, TALDO1 ↑ TOR4A, SPRR3, SLC1A1 ↓ Rate of proliferation ↓	[64]

A vs. B: Relative results of group A group vs. group B. Abbreviations: No., Number; EVs, extracellular vesicles; CRSwNP, chronic rhinosinusitis with nasal polyp; CRSsNP, chronic rhinosinusitis without nasal polyp; sEVs, small EVs; PS, phosphatidylserine; NTA, nanoparticle tracking analysis; WB, western blotting; TEM, transmission electron microscopy; CD, cluster of differentiation; TSG, tumor susceptibility gene; ALIX, ALG-2 interacting protein X; hNEC, human nasal epithelial cell; CST-2, cystatin-2; PAPP-A, pappalysin-A; miR, micro-RNA; GAL10, galectin 10; EPO, eosinophil peroxidase; ITGB5, integrin-β5; EMC4, endoplasmic reticulum membrane protein complex subunit 4; TALDO1, transaldolase 1; TOR4A, torsin family 4 member A; SPRR3, small proline-rich protein 3; SLC1A1, solute carrier family 1 member 1; Ref., reference. ↑: Up-regulation, ↓: Down-regulation.

As shown in Table 2, recently, many studies have been focusing on identifying biomacromolecules altered in individuals with AR, specifically in EVs derived from various biological specimens, compared to those in the normal cohorts. In the nasal mucus, the NMDEs of patients with AR exhibit DEMs, with up-regulated miR-30-5p, miR-199b-3p, and miR-203 levels and down-regulated miR-28-3p, miR-874, and miR-875-5p levels. These DEMs are associated with B-cell receptors and salivary secretion signaling pathways [68]. Li et al. reported low levels of miR-146a-5p in nasal epithelial tissues, NMDEs, and hNECs in patients with AR. As miR-146a-5p promotes T_H_1 differentiation by inhibiting the interaction between mothers against decapentaplegic homolog 3 (SMAD3) and GATA-binding protein 3 (GATA3), its reduced expression is closely related to AR [69]. Similarly, Zhu et al. presented differentially expressed non-coding RNA in patients with AR using both NMDEs and hNEC-derived EVs. Long non-coding RNA GAS5 (lncGAS5) levels are up-regulated in the NMDEs of patients with AR and OVA-treated hNEC-derived exosomes compared to those in the controls [70]. lncRNAs are a subgroup of non-coding RNAs characterized by lengths exceeding 200 nucleotides [71]. Previous studies have partially unveiled the regulatory roles of lncRNAs in influencing the immune system [72]. Increased expression of lncGAS5 induces T_H_2 cell differentiation and AR by decreasing the expression of T-bet and IFN-γ and increasing the expression of GATA3 and IL-4 in CD4^+^ T-cells co-incubated with NMDEs from patients with AR compared to those in the cells co-incubated with NMDEs from controls [70]. As the T_H_1 signature cytokines, including IL-2 and IFN-γ, and T_H_2 signature cytokines are antagonistic toward each other [73], regulation of the T_H_1/T_H_2 ratio is important for AR management [74]. Differential expression of specific molecules in AR has been reported in blood samples. EVs obtained from the plasma of patients with AR exhibit increased expression of the peptide fragments of Der p1—a protease from the house mite Dermatophagoides pteronyssinus—which is notably related to the symptoms of patients with AR and plasma IL-13 levels by inducing the differentiation of T_H_2 cells [75]. The Der p1, which is commonly found in house dust mites, disrupts the epithelial barrier [76]. Therefore, EVs containing Der p1 may be closely related with T2I pathogenesis, especially during antigen presentation.

Unlike the limited research on CRSwNPs, many studies have assessed the potential therapeutic applications of EVs for AR. Mesenchymal stromal cell (MSC)-derived small EVs (sEVs) are promising therapeutic agents for AR. In a previous study, treatment of MSC-derived sEVs delivering miR-146a-5p into ILC2s derived from the peripheral blood mononuclear cells of patients with AR decreased IL-4, IL-5, and IL-13 levels and increased IL-1α, IL-1β, IL-6, and IL-1 receptor antagonist (IL-1RN) levels [77]. This aligns with previous reports that miR-146a-5p levels are decreased in the NMDEs of patients with AR and supports the immunoregulatory effects of MSCs against various immunological diseases [69]. MiR-146a-5p has been identified as a molecule that regulates IL-33 release from ILC2s by inhibiting IRAK1 (IL-1R-associated kinase 1) and TRAF6 (Tumor necrosis factor receptor-associated factor 6), which are key regulators in the IL-33-ILC2 signaling pathway [78]. Therefore, MSC-derived EVs containing miR-146a-5p may have a beneficial effect on AR.

MSC-derived sEVs do not just affect DCs and T-cells; ILC2s ImDCs treated with MSC-derived sEVs exhibit reduced levels of CD11c, CD40, CD80, CD86, and HLA-DR, inhibiting their maturation compared to pre-sEV treated DCs. Furthermore, mDCs treated with these sEVs decrease the counts of IL-4^+^ and IL-13^+^ T_H_ cells and increase the counts of regulatory T-cells (Tregs) by promoting IL-10^+^ T_H_ cells through co-culture with mDCs and CD4^+^ T-cells from the peripheral blood mononuclear cells of patients with AR [79]. Tregs inhibit T_H_2 cell functions by producing IL-10 [80]. These reports suggest that MSC-derived sEVs could be promising therapeutic agents for AR.

**Table 2:** List of studies investigating extracellular vesicles (EVs) in allergic rhinitis (AR).

No.	EV Donor	Used EVs	EV Isolation Methods	EV Identification Methods	Subjects	Results/Effects	Ref.
1	Mucus	Exosomes/sEVs	Centrifugation (3000× *g* for 15 min, 10,000× *g* for 30 min, 50,000× *g* for 1 h, 100,000× *g* for 1 h)	Bead-based flow cytometry (MHCII, CD63)	AR patients vs. Control	miR-30-5p, 199b-3p, and 203 ↑ miR-28-3p, 874, and 875-5p ↓	[68]
2	Nasal mucus	Exosomes/sEVs	Centrifugation (12,000× *g* for 45 min at 4 °C) Supernatant centrifugation (110,000× *g* for 2 h) Filtration with 0.22-μm filter Centrifugation (110,000× *g* for 70 min)	TEM, NTA (10–210 nm), WB (CD9, CD63, ALIX)	AR patients vs. Control	miR-146a-5p ↓	[69]
3	Mucus/Epithelial cell	Exosomes/sEVs	Centrifugation (12,000× *g* for 45 min) Ultracentrifugation (110,000× *g* for 2 h) Filtration with a 0.22-μm filter Centrifugation (110,000× *g* for 70 min)	TEM, WB (CD63, CD81)	AR patients vs. Control OVA treatment vs. non-treatment in RPMI-2650 AR patient EVs vs. Control EVs co-incubated with CD4^+^ T-cell	LncGAS5 ↑ IFN-γ, T-bet ↓ in CD4^+^ T-cells IL-4, GATA3 ↑ in CD4^+^ T-cells	[70]
4	Plasma	Unknown	Ultracentrifugation	NTA, TEM, WB (CD9, CD63, CD81, ALIX, TSG101)	AR patients vs. Control	Der p1 ↑	[75]
5	MSCs	sEVs	Centrifugation (2000× *g* for 20 min) Anion-exchange chromatography Ultracentrifugation (300× *g* for 5 min, 2000× *g* for 20 min, 12,000× *g* for 30 min, 110,000× *g* for 70 min)	Flow cytometry (CD9, CD63, CD81), ELISA (CD63), WB (CD9, CD63, CD81, ALIX, TSG101) Protein concentration, NTA, TEM	EV treatment after vs. before using ILC2s from patients with AR PBMCs	IL-4, IL-5, and IL-13 ↓ IL-1α, IL-1β, IL-6, and IL-1RN ↑ ILC2 function ↓	[77]
6	MSCs	sEVs	Centrifugation (2000× *g* for 20 min) Protein concentration	NTA (85–284 nm) TEM (<150 nm) WB (CD9, CD63, ALIX, TSG101)	EV treatment after vs. before using imDCs EV-treated DCs vs. Control DCs co-cultured with T-cells from patients with AR PBMCs	CD11c, HLA-DR, CD40, CD80, and CD86 ↓ IL-4 and IL-13 ↓ in T-cells IL-10 ↑ in T-cells Treg expansion ↑	[79]
7	Hypoxic-MSCs	Hypoxic-MSC-derived EVs	Ultracentrifugation (100,000× *g* for 90 min)	TEM, NTA, WB (CD63, CD9, TSG101)	Treatment with hypoxic EVs vs. normal EVs of AR mice EV treatment after vs. before using imDCs	VEGF ↑ in hypoxic EVs IL-4, IL-10, mucosa thickness, and inflammation ↓ in nasal mucosa CD40, CD80, and CD83 ↓	[81]
8	hADSCs	Unknown	Medium Centrifugation (300× *g* for 15 min, 4000× *g* for 15 min, 10,000× *g* for 30 min) Ultracentrifugation (130,000× *g* for 90 min)	TEM, NTA (100 nm), WB (CD63, CD81, HSP70)	EV treatment after vs. before in AR mice	Nasal symptoms and inflammatory cell infiltration ↓ IgE, IL-4, and IFN-γ ↓ in serum Ratio of T_H_1/T_H_2 ↑	[82]

A vs. B: Relative results of group A vs. group B. Abbreviations: No., Number; EVs, extracellular vesicles; AR, allergic rhinitis; MSCs, mesenchymal stromal cells; hADSCs, human adipose tissue-derived stem cells; sEVs, small EVs; TEM, transmission electron microscopy; NTA, nanoparticle tracking analysis; WB, western blotting; MHC, major histocompatibility complex; CD, cluster of differentiation; ALIX, ALG-2 interacting protein X; TSG, tumor susceptibility gene; HSP, heat-shock protein; ILC2, type 2 innate lymphoid cell; DC, dendritic cell; imDC, immature DC; mDC, mature DC; PBMC, peripheral blood mononuclear cell; miR, micro-RNA; Lnc, long non-coding RNA; IFN, interferon; IL, interleukin; GATA, GATA binding protein; Der p1, Dermatophagoides pteronyssinus 1; IL-1RN, IL-1 receptor antagonist; VEGF, vascular endothelial growth factor; IgE, immunoglobulin E; T_H_, helper T-cell; Ref., reference. ↑: Up-regulation, ↓: Down-regulation.

The effects of MSC-derived EVs on AR have been demonstrated in vivo using an AR mouse model. Notably, EVs derived from MSCs cultured under hypoxic conditions exert more pronounced therapeutic effects than those cultured under non-hypoxic and pre-treatment conditions. This enhanced effect is attributed to the inhibition of imDC maturation and reduction in *IL-4* and *IL-5* expression, nasal mucosa thickness, and T2I in the nasal mucosa, which are largely mediated by the vascular endothelial growth factor present in EVs [81]. In addition to MSCs, human adipose tissue-derived stem cells (hADSCs) exhibit promising effects against AR. In a previous study, similar to MSCs, hADSCs-derived EVs decreased the serum levels of soluble IgE, IFN-γ, and IL-4, ultimately reducing inflammatory cell infiltration and AR symptoms by enhancing the T_H_1/T_H_2 ratio in an AR mouse model [82].

### 4.3. The Application of EVs for Asthma

Asthma is a complex respiratory condition characterized by inflammation of the lower airway tract, bronchial hyperresponsiveness, and variable airflow obstruction [83]. It is often associated with other complications, such as AR and CRSwNP [6]. Typical symptoms of asthma include wheezing, shortness of breath (dyspnea), chest tightness, and coughing. Asthma is an inflammatory disease exhibiting diverse characteristics and heterogeneous phenotypes and endotypes similar to those observed in CRSwNP. Notably, eosinophilic asthma (eA), predominantly mediated by T2AI, is the most common phenotype/endotype of severe asthma [84].

Many studies have investigated the roles of EVs in asthma (Table 3). Preliminary studies have investigated the effects of IL-13 on normal human bronchial epithelial cells (hBECs) under asthmatic conditions in vitro. One study reported increased exosome secretion in healthy hBECs after IL-13 treatment compared to the pre-treatment levels, facilitating monocyte proliferation and chemotaxis [85]. Another study using healthy primary hBECs reported decreased expression levels of miR-34a, miR-92b, and miR-210 after IL-13 treatment. The target genes of these miRNAs are mainly involved in DC maturation and regulation of T_H_2 differentiation [86]. Additionally, DEMs, such as let-7, miR-9, and miR-10, have been identified in the hBECs of patients with asthma. These miRNAs are primarily associated with TCR signaling pathways, T-cell differentiation, and plasma cell differentiation [87]. These reports suggest that EVs and their miRNA cargos play significant roles in the pathogenesis and progression of asthma by modulating key immune processes.

Another study investigated EVs under in vitro asthmatic conditions using normal hBECs, particulate matter less than 2.5 µm in diameter (PM_2.5_), and blood samples from patients with asthma. The study reported that let-7i-5p levels are significantly up-regulated in both normal hBECs treated with PM_2.5_ and plasma-derived EVs from patients with asthma compared to those in the pre-treatment and control groups, respectively. This miRNA has been linked to juvenile asthma development, as injection of let-7i-5p-loaded EVs into a juvenile asthmatic mouse model aggravated asthma symptoms [88]. EVs derived from the blood of patients with eA exhibit both DEMs and DEPs. Elevated levels of GAL10, EPO, major basic protein 1, eosinophil-derived neurotoxin, and arachidonate 15-lipoxygenase have been reported in the EVs of a patient with eA serum [61]. As mentioned above, GAL10 and EPO are over-expressed in EVs derived from the serum of patients with CRSwNP, indicating their potential as biomarkers and therapeutic targets for individuals with co-existing CRSwNP and asthma.

Several studies have explored the potential therapeutic applications of EVs for asthma. MSC-derived EVs decrease the IL-5 and IL-13 levels, inflammatory cell infiltration in bronchoalveolar lavage fluid (BALF), and ILC2 and goblet cell hyperplasia in lung epithelial cells, ultimately reducing airway hyper-responsiveness in asthmatic mouse models. These effects are associated with miR-146a-5p expression in MSC-derived EVs [77]. Furthermore, EVs derived from hypoxic MSCs, which also contain miR-146a-5p, ameliorate T2I by reducing eosinophil and T_H_2 mediator counts in the BALF of asthmatic mouse models [89]. As miR-146a-5p is downregulated in the EVs derived from the mucus of patients with AR [69], approaches to increase its levels may inhibit T2AI development. In addition to MSCs, ADSCs exert potent effects against asthma. ADSCs-derived EVs decrease the total IgE levels in serum and IL-4 levels in lung-draining lymph nodes and BALF and they increase Treg proportions in the lung-draining lymph nodes of asthmatic mouse models [90]. These findings underscore the potential of EV-based therapies targeting key inflammatory pathways and cellular mechanisms for asthma management.

Effects of EVs on asthma have been investigated using mast cells. Exosomes derived from bone marrow-derived mast cells were found to mitigate airway hyper-responsiveness and inflammation by binding to free IgE on exosomal FcεR in asthmatic mouse models [91]. However, EVs derived from a mouse mast cell line aggravated airway inflammation in an asthmatic mouse model by decreasing the antioxidant enzyme levels and increasing the inflammatory cell counts via the delivery of miR-21, one of the up-regulated DEMs in asthma [92]. These reports indicate the complex effects of mast cell-derived EVs on T2AI, underscoring the need for further studies to analyze their roles and therapeutic potential.

## 5. Conclusions

**Figure 3:**
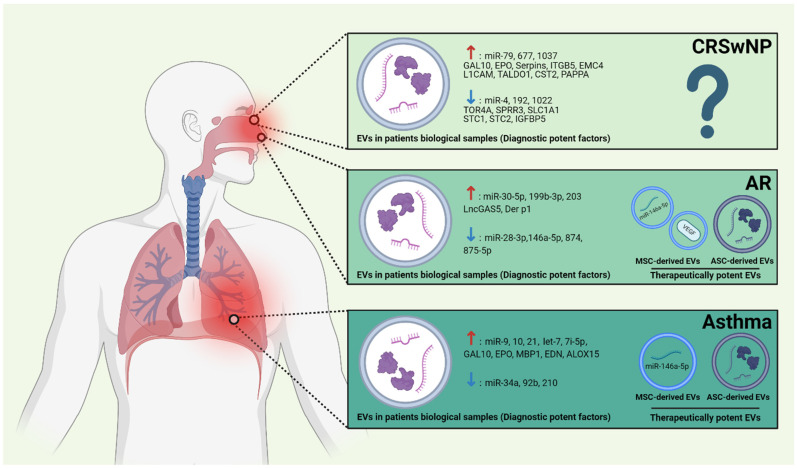
Comprehensive applications of EVs for various T2AI diseases. EVs exhibit differentially expressed molecules in chronic rhinosinusitis with nasal polyps (CRSwNP), allergic rhinitis (AR), and asthma and can be applied for the diagnosis of these conditions. Specifically, mesenchymal stromal cells (MSCs) and adipose tissue-derived stem cells (ADSCs)-derived EVs are potential therapeutic targets for AR and asthma. ↑: Up-regulation, ↓: Down-regulation.

Taken together, this review describes the roles of EVs of various origins in the pathogenesis and progression of the T2AI diseases, CRSwNP, AR, and asthma. Additionally, it highlights their potential applications in the clinical diagnosis and treatment of these conditions. Figure 3 illustrates these potential diagnostic and therapeutic applications of EVs for T2AI diseases. Furthermore, this review provides a concise overview of the methods used to isolate and identify EVs in recent T2AI studies.

Many studies have used EVs of various origins, including patient-derived samples and animal models, to investigate asthma and AR. However, only a few have used EVs to study CRSwNP compared to AR and asthma. Also, verification of EV use is predominantly based on animal models or in vitro experiments. This may be attributed to challenges in the technical detection and isolation of EVs, as well as the reproducibility of EV acquisition, which is complicated by their heterogeneity. Moreover, in clinical studies including T2AI diseases, EVs isolated from patients are able to exhibit significant heterogeneity and reduced reproducibility, largely due to the diverse range of phenotypes and endotypes expressed within the patient population [93] and owing to non-standardized EV isolation and identification methods [94].

Therefore, it is crucial for further studies to focus on rigorously validating the findings from in vitro and animal models through translational medicine, ultimately advancing these results into clinical applications by establishing standard EV isolation and analysis methods and increasing the reproducibility EVs obtain [95]. Consequently, additional clinical studies will be essential to thoroughly evaluating the diagnostic and therapeutic potential of EVs, confirming their specific roles, and accelerating their integration into clinical practice.

## Figures and Tables

**Table 3:** List of studies investigating EVs in asthma.

No.	Donor	Used EVs	EV Isolation Methods	EV Identification Methods	Subjects	Results Effects	Ref.
1	Healthy hBECs (BEAS-2B)	Exosomes/sEVs	Centrifugation Ultracentrifugation	Bead-based assay (HSP70, CD63) WB (CD63, TSG101, ALIX, RAB5B, RAB27A)	IL-13 treatment after vs. before	Exosome secretion ↑	[85]
2	Healthy primary hBECs	Exosomes/sEVs	Basal/apical medium with ExoQuick-TC kit	NTA (5–1000 nm), TEM, WB (CD63, CD9, ALIX), SEC (10 kDa, 0.2 µm)	IL-13 treatment after vs. before	miR-34a ↓ in basal EVs miR-92b ↓ in basal EVs miR-210 ↓ in apical EVs	[86]
3	Primary hBECs	Exosomes/sEVs	Basal/apical medium centrifugation (500× *g* for 10 min, 2000× *g* for 20 min, 10,000× *g* for 30 min) Filtration with 0.22-µm filter, ExoEasy kit	NanoFCM (50–250 nm), Bead-based flow cytometry (CD9, CD63, CD81)	Patients with asthma vs. Control	miR-9 ↑ in apical EVs let-7, miR-9, and miR-10 ↑ in basal EVs	[87]
4	Healthy hBECs plasma	Exosomes/sEVs	Culture of hBECs with ExoQuick TC Kit ExoQuick Plasma Prep with Thrombin Kit	TEM, NanoFCM, WB (CD63, TSG101, ALIX)	PM_2.5_ treatment after vs. before Patients with asthma vs. Control	let-7i-5p ↑	[88]
5	Serum	Exosomes/sEVs	Phosphatidylserine (PS) affinity method	TEM (CD9) NTA (<200 nm)	Patients with eA vs. Control	GAL10, EPO, MBP1, EDN, ALOX15 ↑ in EVs	[61]
6	hMSCs	sEVs	Centrifugation (2000× *g* for 20 min) Anion-exchange chromatography Ultracentrifugation (300× *g* for 5 min, 2000× *g* for 20 min, 12,000× *g* for 30 min, 110,000× *g* for 70 min)	Flow cytometry (CD9, CD63, CD81), ELISA (CD63), WB (CD9, CD63, CD81, ALIX, TSG101) Protein concentration, NTA, TEM	EV treatment after vs. before in asthmatic mice	miR-146a-5p ↑ in MSC-derived EVs Infiltration of inflammatory cells ↓ in peritracheal area and BALF IL-5 and IL-13 ↓ in BALF Epithelial goblet cells ↓ in lungs ILC2 and airway hyperresponsiveness ↓ in lungs	[77]
7	Hypoxic-hMSCs	Unknown	Ultracentrifugation (300× *g* for 10 min, 2000× *g* for 20 min, 100,000× *g* for 90 min)	TEM, WB (HSP70 TSG101), NTA (150–160 nm)	Hypoxic MSC vs. Normal MSC EV treatment after vs. before in asthmatic mice	miR-146a-5p ↑ in hypoxic MSC-EVs Total cells, eosinophils, and T_H_2 mediator ↓ in BALF Airway inflammation ↓	[89]
8	Murine ADSCs	Unknown	Filtration with 0.45 and 0.22-µm filters Ultracentrifugation (100,000× *g* for 2 h)	TEM (100–400 nm), NTA (127 nm), WB (CD40 and CD81)	EV treatment after vs. before in asthmatic mice	Total IgE and IgG ↓ in serum Inflammatory cells, eosinophils, and IL-4 ↓ in BALF Eosinophilic Lung inflammation ↓ IL-4 ↓ lung draining lymph nodes Treg ↑ in lung draining lymph nodes	[90]
9	Murine BMMCs	Exosomes/sEVs	Centrifugation	TEM (50–80 nm), WB (FcεRI), Flow cytometry, Confocal microscopy	Exosome treatment after vs. before in asthmatic mice	Airway inflammation ↓ Airway hyperresponsiveness ↓	[91]
10	Murine mast cell (MC/9)	Unknown	Centrifugation (300× *g* for 10 min, 20,000× *g* for 20 min) Filtration with a 0.2-µm filter Ultracentrifugation (100,000× *g* for 90 min)	TEM, NTA, WB (HSP70, CD9, TSG101)	EV treatment after vs. before in mouse epithelial cell line/asthmatic mice	MiR-21 ↑ in mouse mast cell-derived EVs/asthmatic Mice miR-21 ↑ after MC-EV treatment in mouse epithelial cells Antioxidant enzymes ↓/inflammatory cells ↑ in asthmatic mice	[92]

A vs. B: Relative results of group A vs. group B. Abbreviation: No., Number; EVs, extracellular vesicles; hBECs, human bronchial epithelial cells; hMSCs, human mesenchymal stromal cells; ADSCs, adipose tissue-derived stem cells; BMMCs, bone marrow-derived mast cells; sEVs, small EVs; NTA, nanoparticle tracking analysis; TEM, transmission electron microscopy; SEC, size exclusion chromatography; WB, western blotting; FACS, fluorescence-activated cell sorting; CD, cluster of differentiation; ALIX, ALG-2 interacting protein X; TSG, tumor susceptibility gene; HSP, heat-shock protein; RAB, Ras-related protein; IL, interleukin; PM_2.5_, particulate matter less than 2.5 µm in diameter; eA, eosinophilic asthma, miR, microRNA; GAL10, galectin 10; EPO, eosinophil peroxidase; MBP1, major basic protein 1; EDN, eosinophil-derived neurotoxin; ALOX15, arachidonate 15-lipoxygenase; BALF, bronchoalveolar lavage fluid; Ig, immunoglobulin; Treg, regulatory T-cell; Ref., reference. ↑: Up-regulation, ↓: Down-regulation.

## Data Availability

Data used in this study are available upon reasonable request from the corresponding author. All figures included in this article were created with Biorender (BioRender.com).
